# Occurrence of microplastics in wild oysters (*Crassostrea tulipa)* from the Gulf of Guinea and their potential human exposure

**DOI:** 10.1016/j.heliyon.2022.e12255

**Published:** 2022-12-10

**Authors:** Samuel Addo, Charles Mario Boateng, Rhoda Lims Diyie, Collins Prah Duodu, Anyan Kofi Ferni, Ernestina Abbew Williams, Akosua Ohemaa Amakye, Obed Asamoah, Harriet Danso -Abbeam, Elvis Nyarko

**Affiliations:** a Department of Marine and Fisheries Sciences, School of Biological Sciences, University of Ghana, P. O. Box LG 99. Accra, Ghana; b Council for Scientific and Industrial Research, Water Research Institute (CSIR-WRI), P. O. Box AH 38, Accra, Ghana; c University of Development Studies, P. O. Box TL 1350, Tamale, Ghana; d Environmental Resources Research Centre, Ghana Atomic Energy Commission, Post Office Box LG 80, Legon, Accra, Ghana; e Regional Maritime University, Post Office Box GP 1115, Accra, Ghana

**Keywords:** Microplastics, Polymer, Fibers, Oysters, Human exposure

## Abstract

The high dependence on plastics in Ghana has resulted in the generation of large quantities of plastic waste which are poorly managed and improperly disposed into the aquatic environments. This study assessed the spatial distribution and abundance of microplastics in mangrove oysters (*Crassostrea tulipa*): a major fishery resource of commercial importance in Ghana. The results showed that 84.0% of all individuals examined had ingested microplastics. A total of 276 microplastic items were recovered from the 120 individual oysters. Densu (100%) and Volta (93%), two estuaries situated in urban areas, had a greater incidence of microplastics than Whin (77%) and Nakwa (66%), estuaries situated in peri-urban and rural settlements, respectively. The mean microplastic abundance ranged from 1.4 to 3.4 items/individual and 0.34 to 1.7 items/g tissue wet weight. Fiber accounted for 69% of microplastic shapes, followed by fragments (27%) and films (4%). Polymer analysis showed polyethylene (PE), polypropylene (PP) and polystyrene (PS) as the most common types in oysters. The estimated microplastic intake per capita per year was one magnitude higher than the mean for other countries. This high rate of human exposure to microplastics requires an eminent policy formulation to guide the use, management and disposal of plastic waste in Ghana.

## 1 Introduction

Global plastic waste generation threatens food security and the sustainable utilization of marine resources (Campanale et al., 2020), as developing countries with poor waste management strategies are more likely to face severe impacts on human health and the environment (Alpizar et al., 2020). Over the past few decades, Ghana has rapidly expanded socio-economically, which has coincided with a significant rise in the use of plastic products (Ghana National Plastic Action Partnership, 2021). The majority of the 840 thousand tonnes of municipal plastic waste generated annually in the nation which is growing at a rate of 5.4% each year comes from single-use plastics. This is exacerbated by the country's poor waste management alternatives, which result from a low recycling rate and a high proportion of illicit disposal (Effah, 2019; Kortei and Quansah, 2016; Stoler et al., 2015). According to predictions made in the Ghana National Plastic Action Partnership (2021) report, for instance, the amount of plastic that leaks into the nation's water bodies is expected to rise by 190%, from about 78,000 tonnes per year in 2020 to 228,000 tonnes per year by 2040. Under suitable biophysical and environmental conditions, the majority of the plastic waste in terrestrial and aquatic systems disintegrates into small particles called microplastics (plastic particles from 1 μm to 5 mm) (ISO, 2020), which could be easily ingested by organisms such as invertebrates and fish (Wang et al., 2021).

Coastal ecosystems particularly estuaries are sensitive to environmental pollution and may serve as conduits for microplastic transfer and accumulation. Plastic materials may enter estuaries from rivers through spills, runoff, sewage discharges and municipal waste treatment plants (Wagner et al., 2014). Several studies have reported significant occurrences of microplastics in the estuarine environment compared to nearshore waters affirming its role in plastic pollution (Peng et al., 2017; Zhao et al., 2014). Hence, estuaries have become the most critical path for microplastic transport to the ocean. Lebreton et al. (2017) reported that between 1.5-2.4 million tonnes of macro and microplastic litter enter the sea through estuaries worldwide. Therefore, studies on the microplastic accumulation in estuarine organisms are important to understand their spatial distribution and transfer into the ocean.

Oysters are good bioindicators of aquatic pollution because their unique feeding strategy allows them to filter large amounts of water through their gills, thereby exposing the organisms to filamentous microplastics found in aquatic media (Wang et al., 2021, Wang et al., 2021; Teng et al., 2019). The mangrove oyster is a popular seafood choice in West Africa and may be found in many of the region's estuaries and lagoons. The mangrove oyster fisheries is a lucrative industry in which women who pick and process the catch play a disproportionately large role (Asare et al., 2019). However, these organisms are often exposed to microplastic pollution from human activities through the improper disposal of plastic refuse near rivers and estuaries in Ghana. The prospect of oyster culture within the West African region is not well exploited, thus, forcing consumers to rely solely on oysters harvested from the wild. Currently, shellfish consumption is the primary route of human dietary exposure to microplastics (Vital et al., 2021).

Several studies have reported microplastic ingestion and toxicity in marine organisms from various ecosystems worldwide (Lerebours et al., 2022; Said et al., 2022; Zhang et al., 2022; Wang et al., 2021a; 2021b; Li et al., 2018; Martinelli et al., 2020; Patterson et al., 2019; Teng et al., 2019; Bour et al., 2018; Digka et al., 2018; Li et al., 2018; Murphy 2018; Renzi et al., 2018 Avio et al., 2015, Cauwenberghe et al., 2015; De-Witte et al., 2014) with little information on their impact on human health when consumed. Nonetheless, the extent of exposure and toxicity may vary depending on environmental conditions and geographic location. Microplastics may contain endocrine-disrupting chemicals (Bisphenol-A) which are normally added as additives during the polymerization process of plastic production or serve as receptors for absorbing contaminants such as Polychlorinated biphenyls (PCBs), Polybrominated biphenyl ethers (PBDEs) and trace metals in the environment (Gallo et al., 2018). These chemicals may leach into edible tissues of the marine organism through translocation, increasing human exposure *via* the consumption of contaminated seafood products (Fossi et al., 2012). Again, most people consume whole oysters including the stomach content which may expose consumers to microplastic intake, while the digestive tract of larger finfishes which happens to be the repository for microplastics is discarded during ingestion.

Despite reports of microplastic pollution in Ghana from Blankson et al. (2022), Pappoe et al. (2022), Nuamah et al. (2022), Adika et al. (2020), and Chico-Ortiz et al. (2020), there is a paucity of information on the occurrence, distribution, and accumulation of microplastics in seafood particularly oysters from the coastal waters of Ghana and their potential human health risk. Therefore, this study assessed the abundance and spatial distribution of microplastics in the tissue of oysters and categorized them into colour, size, shape and polymeric type. In addition, the potential human exposure through the consumption of microplastic-contaminated oysters was estimated.

## 2 Methodology

### 2.1 Study area

**Figure 1:**
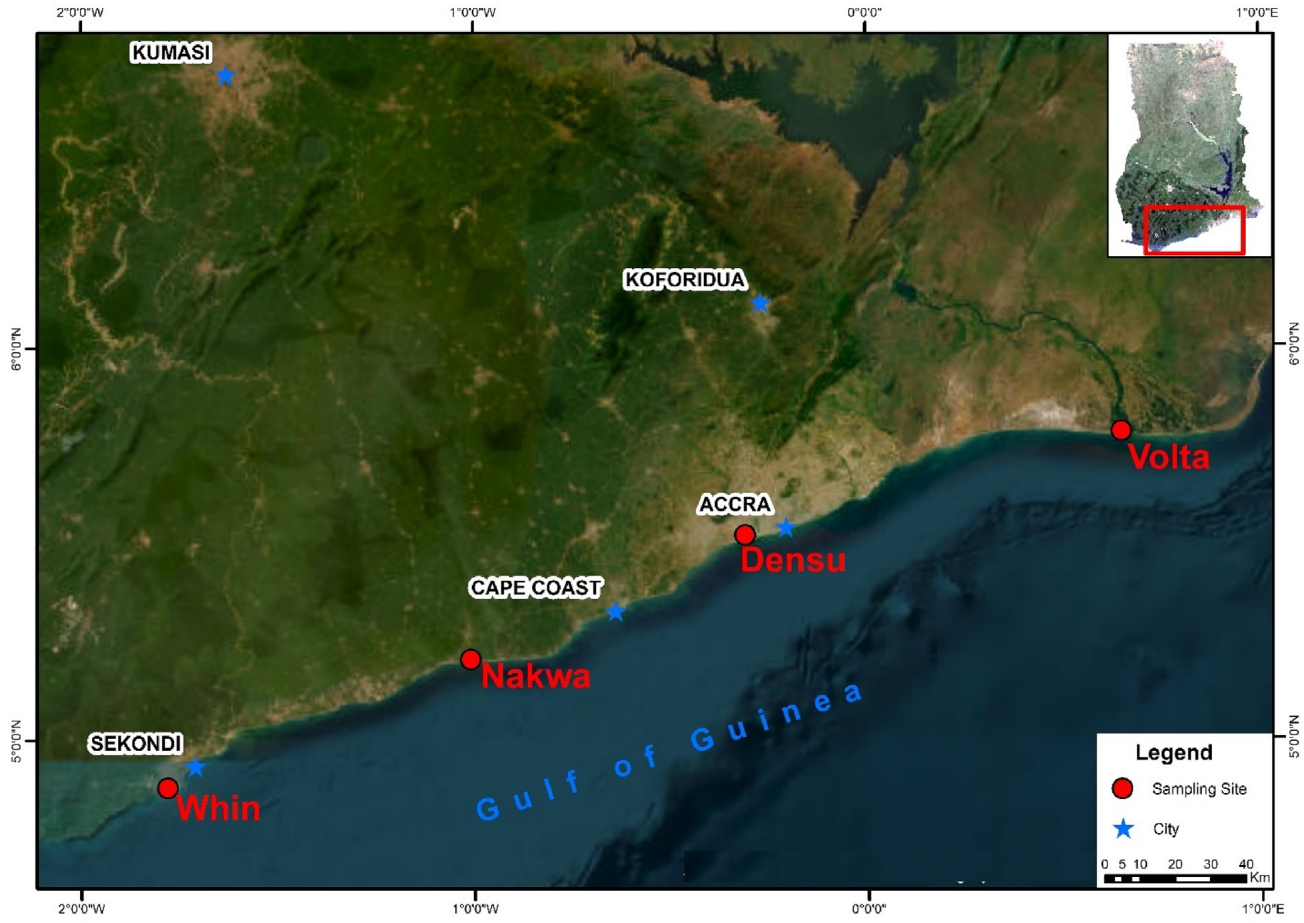
Map of Ghana showing the location of the four water bodies along the coast of Ghana.

Live oysters were collected from Volta, Densu, Nakwa and Whin estuaries along the coast of Ghana from March to April 2021 (Figure 1). The selection of these sites was based on their vibrant oyster harvesting activities. The Volta River flows through a distance of about 670 km from the northern to the southern part of Ghana with its lower arm towards the estuary becoming a hub for tourism yet provides over 60% of inland fish catch (Nunoo et al., 2014). The Densu River flows from the Atiwa forest in Ghana's Eastern Region and spans about 120 km, passing through over 300 communities and industries. Industrial activities include a water treatment plant that supplies potable water to about 4.8 million Ghanaians. Due to its ecological importance, the Densu estuary is designated as a RAMSAR site despite the influx of anthropogenic activities such as mining and intensive agriculture within its buffer zone, coupled with rapid urbanization (Kasei et al., 2014). The Whin estuary is located in the Western Region of Ghana and provides livelihood support through finfish and shellfish trade to the surrounding communities. The Nakwa estuary, a 3.6 square kilometer water body in the Central Region of Ghana along the coast, is a shallow water body adorned with mangroves, and extensive swaths of *Paspalum vaginatum* marshlands.

### 2.2 Sample collection

Mangrove oysters were collected randomly from each estuary with the assistance of oyster pickers. A total of one hundred and twenty (N = 120) individuals (30 per site) of varying sizes were collected and placed in a locally manufactured metal case. Samples were washed thoroughly with distilled water to remove sand particles and transported on ice to the laboratory where they were stored at –20 °C until analysis.

### 2.3 Microplastic extraction

Prior to the extraction of microplastics, the oyster samples were thoroughly thawed at room temperature and the shell length and height were measured with a vernier calliper. The shells were opened from the posterior end, and the muscle was shucked out with a stainless-steel knife into a clean glass beaker. The soft tissue was weighed using a precision balance (Philip Harris, England) to the nearest 0.01 g. Microplastic extraction was carried out in accordance with the protocol by Hara et al. (2020), Phuong et al. (2018), and Karami et al. (2017) with minor modifications. The whole oyster tissues were digested separately with 30 mL of 10% KOH (analytical grade). The digested sample was covered with aluminium foil and incubated at 60 °C for 48 h. Density separation was performed three times to enhance the maximum recovery of microplastics after incubation by the addition of 150 mL saturated ZnCl_2_ solution (1.6 g/cm^3^) and agitated for 10 min with a mechanical shaker (Rodrigues et al., 2018; Zobkov and Esiukova 2017). The mixture was allowed to stand undisturbed for 3 h at room temperature, and the supernatant was filtered through a 1.2 μm glass filter (CHMLAB GF3) using a vacuum filtration system. The glass filters were then placed in a clean Petri dish, dried at 40 °C for 60 min, sealed, and kept in a desiccator for examination. Procedure blank samples, consisting of the extraction solution (10% KOH) without samples, were analyzed in parallel with the digested fish samples. Such blanks were necessary to evaluate any potential contamination from the laboratory atmosphere that might have happened during digestion procedures despite all precautions being taken.

### 2.4 Microplastics examination

Filters were examined and counted for microplastics with Stereomicroscope (Leica EZ4D) equipped with an inbuilt camera (Leica ICC550E). To avoid overestimation, the filters were examined in a Z-shape, according to the method by (Teng et al., 2019; Peng et al., 2017). All plastics were separated and photographed to determine their size and shape using the Spotter Guild protocol developed by the Civil Laboratory for Environment Action Research (CLEAR) for microplastic identification and shape guide by Frias et al. (2019). The shape of the plastics was categorized as fragments (irregular and angular pieces mainly from the degradation of large plastic materials), fibers (long and elongated), pellets (spherical shapes) and film (thin and transparent). The colours of microplastics were classified into black, blue, white, red, yellow and others according to Edo et al. (2019). Size categorization was based on the recommendation by Frias and Nash (2019).

### 2.5 Polymer identification

Fourier Transform Infrared Spectroscopy was used to identify the polymer (FT-IR). Background scans were performed before analysis to account for any potential distortions in the data. Eighty isolated microplastics (20 per estuary) were then examined using an FT-IR microscope (PerkinElmer Spotlight i200) and a spectrometer (Spectrum Two, PerkinElmer). Using spectrum 10 software, each sample was subjected to 16 scans at a resolution of 4 cm^−1^. The OMNIC v9.0 spectra software (Thermo Fisher Scientific Inc.) was used to evaluate the output spectra and compare them to its polymer libraries. Finally, reference spectra ranging from 70 to 95 per cent were used to match polymers.

### 2.6 Quality control

The extraction of microplastics was done in a cleanroom where workbenches were cleansed with distilled water before and after each phase of the extraction procedure. The dissection of tissues was done in a fume hood. During oyster removal, glass and metal items were utilized whenever possible and were cleaned with distilled water before use. Super-pure reagent-grade chemicals were used to prepare the extraction solution. The prepared solution for digestion and density separation was filtered through a 0.45 μm glass filter (CHEM-LAB- A55). Wearing of synthetic clothing was limited throughout the entire process. All glassware was covered with aluminum foil to prevent airborne contamination from the working environment. Contamination controls consisted of a sample-free KOH solution that was subjected to all the extraction processes in the same conditions as the samples. Glass fiber filter was heated in a muffle furnace for 3 h at 450 °C to eliminate any contamination for the filters.

### 2.7 Human microplastic exposure via oyster consumption

Human health risk exposure to microplastics via the consumption of contaminated oysters was determined using a simple parametric approach. The first assessment was to estimate the weekly intake of MPs items using the recommended shellfish consumption limit per week (50 g/person/week) for Ghana reported by Agbekpornu (2021). The second method was based on data from various countries regarding shellfish consumption per capita per year: Ghana i.e., 2600 g/person/year (Agbekpornu, 2021), France 672 g/person/year (AFSSA, CREDOC DGAC 2000), New Zealand 985.5 grams/person/year (Greening et al., 2003) and USA 3066 g/person/year (OEHHA 2001). Human intake of microplastic from oysters was computed by multiplying the total number of microplastics in oysters from all four estuaries (i.e., the mean total number of microplastics found in oyster tissues of 101 individuals) by the shellfish consumption rate for the various countries (Barboza et al., 2020).

MP intake per week = The mean plastics (g/tissue) in all individuals analyzed × recommended shellfish intake per week (g)

Human MP intake per year per capita = Mean microplastic (g/tissue) in all oysters examined × consumption of shellfish per year per capita in selected countries.

### 2.8 Statistical analysis

Statistical analyses for data generated were performed using IBM SPSS Statistics v26 software. Microplastic abundance was estimated and reported as the number of items per individual. Kruskal-Wallis/One Way Analysis of Variance (One-Way ANOVA) was performed to test for differences in the number of MPs in oysters followed by post hoc Dunn's test for multiple comparisons between the estuaries. Pearson correlation analysis was performed to test the correlation between microplastic abundance and oyster size. The level of significance was set at p < 0.05, and average values were expressed as mean ± standard error of the mean (SE).

## 3 Results

### 3.1 Abundance of microplastics in oyster *C. tulipa*

According to the results obtained, no microplastics were detected in blank samples. The absence of microplastic in the procedural blank samples and filters is both an indication of the accuracy of the laboratory tests and a confirmation of the precision and dependability of the approach that was utilized in the process of recovering the microplastics. The morphometric characteristics of the oysters and site descriptions of the different estuaries where samples were collected are presented in Table 1. The mean weight (g) of oysters was highest for Whin (10.60 ± 1.24) and lowest for the Volta estuaries (4.69 ± 0.82). Meanwhile, shell length ranked as follows: Whin > Densu > Nakwa > Volta (Table 1).

**Table 1:** Morphometry of oysters and site description of estuaries.

Location	Average Shell length (cm)	Average Weight (g)	Site Description
**Densu**	7.20 ± 1.83	5.45 ± 1.54	Urban
**Volta**	4.69 ± 0.82	2.12 ± 0.75	Urban
**Nakwa**	7.01 ± 0.85	3.58 ± 0.84	Rural
**Whin**	10.60 ± 1.24	10.21 ± 1.41	Peri-urban

A total of 276 particles were extracted from the tissues of 120 *C.* *tulipa*, from four estuaries along the Gulf of Guinea with an average of 2.30 ± 1.40 MP items per individual. Of these samples, 101 out of 120 individuals examined (c. 84%) had ingested at least one MP particle. All oysters collected from various estuaries exhibited a distribution profile of microplastics that generally showed a decreasing concentration with distance from metropolitan centers. For instance, a significantly higher abundance of microplastics was observed in samples from Densu (3.4 ± 1.0 items/individual) and Volta (2.8 ± 1.1 items/individual) estuaries near metropolitan areas of Greater Accra, whereas estuaries located near rural settlements contained fewer microplastics, i.e., Nakwa (1.4 ± 1.3 items/individual) and Whin (1.6 ± 1.2 items/individual) (p < 0.01).

**Figure 2:**
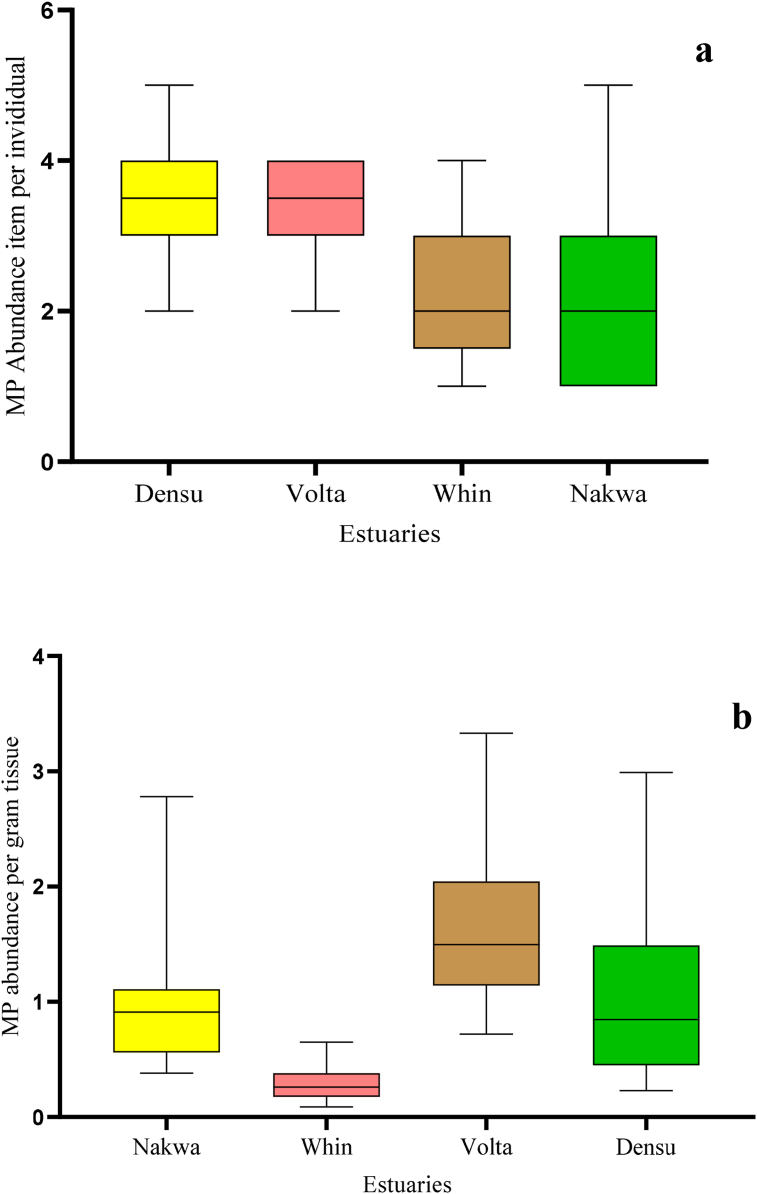
Boxplot showing the range in abundance of MPs extracted from *C. tulipa* at each estuary computed based on (a) items per individual, (b) items per gram tissue (n = 30; N = 120). Boxes represent the first and third quartile, middle bar, the median and error bar maximum and minimum valves.

The percentage of MP occurrence for each estuary is presented in Table 2, where oysters from the Densu delta and the Volta recorded the highest percentage of individuals with MP's (100% and 93% respectively), while the lowest recorded was at the Nakwa estuary (67%). The abundance of MPs ranged from 1 to 5 items per individual, with the highest abundance recorded from the Densu delta (n = 101) and the lowest recorded at the Nakwa estuary (n = 41). Analysis of variance (ANOVA) showed significant differences between microplastic abundance and locations. This significant variation according to the post hoc Dunn test existed between Densu and Nakwa (p < 0.01), and Densu and Whin (p < 0.01). Volta and Nakwa (p < 0.01), Volta and Whin (p < 0.01). Densu and Volta were significantly different from Whin and Nakwa. When samples were examined based on wet weight, oysters collected from the Volta estuary exhibited the highest MP abundance, with an average of 1.64 ± 0.63 items per gram tissue, while the lowest abundance was recorded in the Whin estuary with 0.29 ± 0.14 items per gram tissue (Figure 2b). Meanwhile, a weak correlation was observed between the abundance of microplastic and oyster size from the different locations. Nakwa (r = 0.084, p = 0.766) Whin (r = 0.338, p = 0.178) Volta (r = -0.157, p = 0.533) Densu (r = 0.011, p = 0.965).

**Table 2:** Frequency of occurrence and mean abundance of microplastic in oysters from estuaries in Ghana.

Description	Densu	Volta	Nakwa	Whin
Number of individual oysters Examined	30	30	30	30
Number of individuals containing plastic	30	28	15	17
MP frequency of Occurrence (%)	100	93	67	77
MP Number	101	85	41	49
**MP Abundance**				
Number of items per individual in all examined oysters	3.4 ± 1.0	2.8 ± 1.1	1.4 ± 1.3	1.6 ± 1.2
Number of items per individual in positive oysters	3.4 ± 1.0	3.0 ± 0.8	2.1 ± 1.1	2.1 ± 0.9

### 3.2 Characteristics of microplastics in *C. tulipa*

#### 3.2.1 Shape

**Figure 3:**
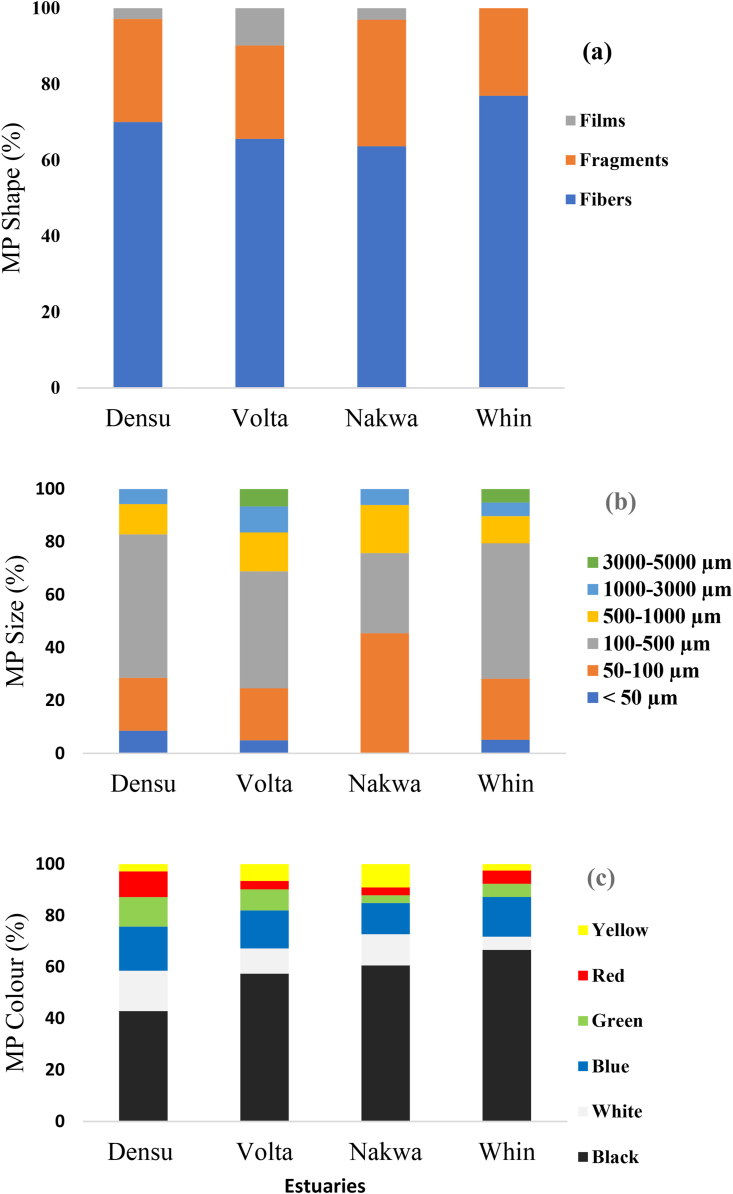
Morphological characteristics of MPs in *C. tulipa*, (a) shapes, (b) sizes, (c) colour.

The proportion of different shapes of microplastics in the sampled specimen is presented in Figure 3a and categorized as: fibers, fragments, and films. Fibers and fragments were found in samples from all studied sites whereas no film was recorded in samples from the Whin estuary. Fiber was the most predominant shape in oysters and accounted for approximately 69% of microplastics identified, while fragments and films recorded 27% and 4%, respectively at the different studied sites. Oysters from Whin (77%) and Densu (70%) recorded the highest percentage of fiber occurrence, while the lowest recorded was Nakwa (64%) (Figure 3a).

#### 3.2.2 Size and colour

The length of MPs ranged from 33 μm to 4870 μm, with <1000 μm being the most common size observed and accounting for more than 90% of all categories (Figure 3b). Specifically, as illustrated in Figure 3b, the proportion of the various sizes for all sampling sites was as follows: 33–50 μm (5.42%), 50–100 μm (24.63%), 100–500 μm (46.80%), 500–1000 μm (13.30%), 1000–3000 μm (6.90%), 3000–5000 μm (2.96%). The results showed that 100% of the extracted MPs were within the defined MP size range (1 μm–5 mm). Microplastics in oysters were in multiple colours, mainly white, green, yellow, blue, black, and red (Figure 3c). Black was the most dominant colour, which accounted for 55% of the total number of microplastics observed. The proportion of deep colours (black, red, and blue) was 68% significantly higher than 24% of light colours (white, yellow, and green) (p < 0.05).

### 3.3 Polymer composition of microplastic

**Figure 4:**
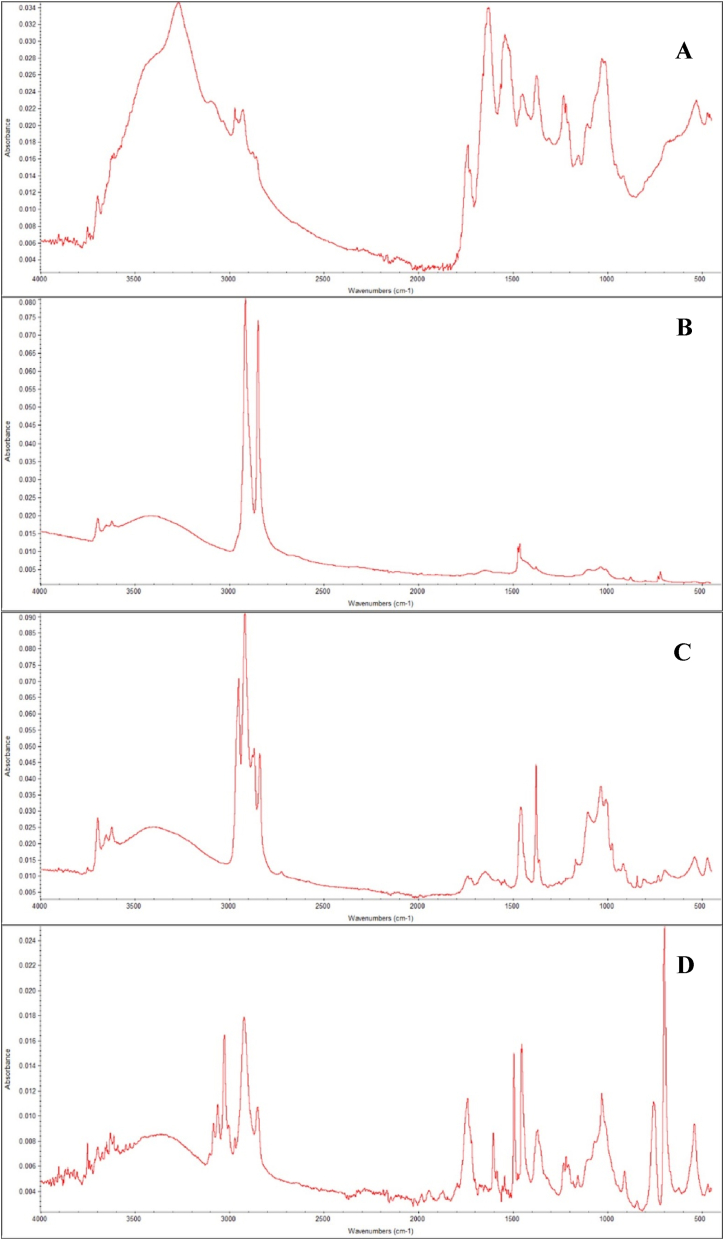
FTIR spectra of common MPs in Oyster. (A) Polyamide (PA), (B) Polyethylene (PE), (C) Polypropylene (PP), and (D) Polystyrene (PS).

Eighty random subsamples of MPs (29 % of the total MPs identified) were selected for FTIR analysis, and 92% of them were found to be synthetic polymers correspondingly. The remaining 8% came from natural sources, principally consisting of fibers of 6% cotton and 2% cellulose Figure 4. Among the synthetic polymers identified, the distribution was ranked as polyethylene (36%), polypropylene (22%), polyamide (16%), polystyrene (12%), cellophane (7%) and polyester (7%). Polyethylene, polypropylene, polystyrene, and polyamide were found to be the most abundant polymers in *C. tulipa* tissues throughout all four estuaries, and together they accounted for 86% of all MPs polymers examined. Other polymers identified were cellophane (CP), cotton and cellulose. See photographs of spectra pecks from FTIR analysis (Figure 4).

### 3.4 Human microplastic intake

Microplastics are inadvertently consumed by humans through the consumption of food derived from a wide variety of sources, and bivalves are not an exception. The study preliminary investigated the weekly and annual oyster consumption for adult populations in Ghana and other regions of the world. In Ghana, the estimated weekly intake for adults ranged from 14.0 to 82.0 MP items per week and 754 to 4160 items per capita per year (Table 3). Based on this backdrop as per their consumption, persons based in the USA, France and New Zealand exposures ranged from (17–94), (1.2–20.8) and (5.5–30.4) for weekly intakes and (889.4–4906), (195–1075) and (286–1577) annually.

**Table 3:** Estimated weekly and annual intake of microplastic for adult populations.

Exposure	Ghana	USA	France	New Zealand
*Weekly intake*				
Oyster consumption/week (g)	50	59	13	19
MPs ranges	14.5–80	17–94.4	1.2–20.8	5.51–30.4
Mean MP intake (item/week)	50	59	13	19
*Annual intake*				
Oyster consumption/capita/year (kg)	2,600	3066	672	985.5
MPs ranges	754–4160	889.4–4906	195–1075	286–1577
Mean MP intake (item/capita/year)	2,600	3066	672	985.5

## 4 Discussion

The study revealed high variability in the spatial distribution and abundance of MP in oysters from the coastal waters of Ghana. The heterogeneous distribution pattern for MP abundance may be due to the unique anthropogenic pressures experienced by each sampled site, the proximity to human settlements, and other commercial activities. For instance, microplastics from urban estuaries were higher than those from rural estuaries, indicating that wastewater from urban cities might be the main source and retention of microplastics. Such observation was made elsewhere by Pappoe et al. (2022); Joyce et al., 2022; Li et al. (2018), where anthropogenic factors from cities played a significant role in microplastic abundance and distribution. The Densu estuary is a marine protected area due to its ecological significance but recorded the highest mean abundance of MPs (Table 2). This could be attributable to the recent encroachment and expansion activities within its buffer zone, which have led to the construction of large-scale commercial activities such as the establishment of industries, hotels, and residential facilities that discharge their waste straight into the estuary. The worse of these is the establishment of the Oblogo dump site within proximity of the water banks coupled with other unauthorized dump sites created by communities dotted along the estuary which serve as key sources of plastic pollution for the Densu river (Osei et al., 2011; Nyame et al., 2012). Microplastic abundance from densely populated and industrialized settlements is projected to be higher than that from rural settlements characterized by minimal human activities. Therefore, it is not surprising that oysters from the Densu and Volta estuaries, located in the Greater Accra Region, Ghana's most populous region recorded more microplastic abundance.

The average abundance of MPs in the current study was 2.5 ± 1.3 items/individual. When compared with shellfish from other regions of the world, the MP abundance in the oysters (*Crassostrea tulipa)* was higher than that in *Crassostrea gigas* from France (Phuong et al., 2018), USA (Martinelli et al., 2020) and South Korea (Cho et al., 2019), as well as *Mytilus edulis* from Belgium (Mathalon and Hill, 2014). The abundance of microplastics found in this study is consistent with those reported in *Perna canaliculus* from New Zealand (Webb et al., 2019) and *Mytilus edulis* from Scotland (Courtene-jones et al., 2017). However, our results are lower than those found in *Crassostrea gigas* from China (Teng et al., 2019), *Magallana bilineatan* from India (Petterson et al. 2019), *Crassostrea gigas* from Canada (Murphy, 2018) and *Crassostrea viginica* from USA (Waite et al., 2018) (Table 4). The variations in MP abundance could be attributed to the extent of plastic contamination of oyster habitats, pollution status and exposure levels of the different geographical areas. Besides, pollution status variability due to locations, and methodological techniques used could also be sources of heterogeneity and variability in the results found in literature.

**Table 4:** Microplastic abundance is shellfish from different parts of the world.

Country	Species	Analytical Method	Microplastics Abundance	Common size (um)	Dominant shape	Dominant polymer	Reference
Ghana	*Crassostrea tulipa*	KOH/ZnCl_2_	2.5 ± 1.3 Item/individual	100–500	Fiber	Polyethylene	This Work
China	*Saccostrea cucullata*	KOH	1.7–7.0 Item/individual	20–50	Fiber	Polyethylene terephthalate	Li et al., (2018)
Greece	*Mytilus galloprovincialis*	H_2_O_2_	0.9 ± 0.2	100–500	Fragment	polyethylene	Digka et al., (2018)
China	*Scapharca subcrenata*	H_2_O_2_/NaCl	10.5 Item/gram	5–250	Fiber	Polyethylene	Li et al., (2015)
China	*Crassostrea gigas*	KOH/H_2_O_2_	2.93 Item/individual	>500	Fiber	Cellophane	Teng et al., (2019)
India	*Magallana bilineata*	KOH/NaI	6.9 ± 3.8	250–500	Fiber	Polyethylene	Petterson et al. 2019
Belgium	*Mytilus edulis*	NHO_3_: HCl	0.35 Item/gram	1000–1500	Fiber	N/A	De Witte et al., (2014)
Belgium	*Mytilus edulis*	HNO_3_	0.2 ± 0.3	20–90	N/A	N/A	Cauwenberghe et al., 2015
Italy	*Mytilus galloprovincialis*	H_2_O_2_	3.6–12.4 Item/individual	1700–1900	Fiber	N/A	Renzi et al., (2018)
New Zealand	*Perna canaliculus*	HNO_3_	0–1.5	100–200	Fragment	Polyethylene	Webb et al. (2019)
USA	*Siliqua patula*	KOH/NaCl	8.8 ± 0.4	500–1000	Fiber	Polyethylene terephthalate	Baechler et al. (2020)
United Kingdom	*Mytilus edulis*	H_2_O_2_/NaCl	1.1–6.4	5–250	Fiber	Polyester	Li et al. (2018)
France	*Crassostrea gigas*	KOH/NaCl	0.60 ± 0.56	50–100	Fragment	Polypropylene	Phuong et al. (2018)
Scotland	*Mytilus edulis*	Enzyme: trypsin	1.05–4.44 Item/gram	1200	Fiber	Polyamide	Courtene-jones et al., 2017
Italy	*Mytilus galloprovincialis*	HNO_3_: HCl	0.11 ± 0.12	N/A	Fragment	N/A	Vandermeersch et al., 2015
USA	*Crassostrea gigas*	H_2_O_2_	0.69–3.00	20–1300	Fiber	Polyethylene	Martinelli et al., (2020)
USA	*Crassostrea viginica*	KOH	3.84 ± 3.39 Item/gram	N/A	Fiber	Polyethylene	Waite et al., (2018)
Canada	*Crassostrea gigas*	HNO_3_	39 ± 27 Item/individual	<530	Fiber	Polystyrene	Murphy 2018
Canada	*Crassostrea gigas*	KOH	0.22 ± 0.22 Item/individual	10–5000	Fiber	N/A	Covernton and Id (2019)
South Korea	*Crassostrea gigas*	KOH	0.97 ± 0.74 Item/individual	100–200	Fragment	Polyethylene	Cho et al. (2019)

The size of microplastics has been found to have a direct association with the frequency of ingestion in bivalves. Smaller-sized MP fractions are more likely to absorb environmental pollutants which may expose human susceptibility to environmental contaminants via shellfish consumption According to the findings of a review study conducted by Fu et al. (2020), it was found that out of 24 publications that were investigated, more than 70% of authors had reported that the dominant microplastic size was less than 1000 μm in aquatic organisms. These findings are comparable to our studies, where approximately 85% of microplastic was discovered in the size range of 50 μm–1000 μm. There is documented evidence that MPs smaller than 20 μm could infiltrate and translocate in the tissues of most marine biota (Hale et al., 2020). Other scientific studies argue that particles of sizes less than 150 μm are expected to be able to pass the human gut barrier and cause adverse effects on sensitive organs. (Chain, 2016). In recent studies, different MP size ranges have been found in human samples. In human placenta samples, for example, MPs identified range in size from 20.3 to 307.3 um, with the 20–100 um size class accounting for 82% of the total MPs detected (Zhu et al., 2022). Amato-Lourenço et al. (2021) reported a size range of 8.12–16.8 μm MP in human lungs, whereas microplastic ranging between 44–210 μm have been found in human sputum (Huang et al., 2022). Comparing these reported sizes in human studies with our findings, we contend that approximately 30% of the microplastics found in the oysters have the potential to pass through human gut barriers when ingested and be translocated to other human organ tissues.

In terms of MP shape, fibers and fragments were the most prevalent microplastics in this study due to their abundance and extensive distribution in the estuarine environment as they have been identified as the most common microplastic in lakes, rivers, estuaries, the ocean, and wastewater treatment plant effluents (Salvador Cesa et al., 2017). Due to their abundant presence in waterbodies, they are more likely to be ingested by invertebrates such as oysters, mussels, shrimps and echinoderms (Baechler et al., 2020; Hermabessiere et al., 2019; Mohsen et al., 2019; Patterson et al., 2019; Li et al., 2015; Vandermeersch et al., 2015) which could impair their feeding behaviour and disrupt their energy budget (Lusher et al., 2013). The sources of these fibers in aquatic environments have been linked to the growing textile manufacturing and hospitality industries along major rivers and lakes that discharge directly into them (Joshy et al., 2022; Browne et al., 2013). However, it is important to note that all the sampled locations in this study are active fishing sites where the loss of synthetic fishing gears (i.e., nets, ropes, and lines) could contribute to fibrous pollution.

The prevalence of black-coloured microplastics may be ascribed to the indiscriminate littering and disposal of polyethylene carrier bags commonly used for shopping in Ghana. Similar results were obtained by Adika et al. (2020) who observed the dominance of black colour among several colours identified as dark coloured microplastics may not be only visible to the oysters but be more prevalent to be ingested due to similarities to prey items and hence more likely to be detected in the oysters as previously observed in experimental studies on fish species (Xiong et al., 2019; Ory et al., 2018).

The polymer types identified in the current studies (polyethylene, polypropylene, polyamide and polystyrene) are principal components of most fishing gears such as ropes, nets, and floats (Pappoe et al., 2022). These fibers may have been accessible to the organisms either because of wear and tear while the fishing gear was being used or possibly as a result of the dangers posed by abandoned, lost or discarded fishing gear, which does not appear to attract the attention of waste managers. Aside its use in the fishing industries, polyethylene and polypropylene are principal components of food packaging, plastic containers and the production of textiles (Joshy et al., 2022; Gedik and Eryaşar, 2020).

The presence of microplastics in the oysters does not only mirror the contamination status of these estuaries in Ghana but also presents a public health challenge to consumers, and hence research on human health effects of microplastics is vital. Although the subtle effect of plastic ingestion on humans is not well understood, there is documented evidence of physical damage to sensitive organs via plastic ingestion (Li et al., 2018). The exposure assessment in this present study indicates that Ghanaian consumers may be exposed to as high as 80 microplastics per week and 4,160 microplastic particles per year (Table 3). Considering the probability of exposure in other regions of the world, estimated annual ingestion rates were highest for the USA followed by New Zealand and France corresponding to the dietary oyster intake rate (Table 3). Although not a common practice for bivalve consumption, in areas where attempts are made to remove the digestive tract, exposures could be minimized.

Based on the average MP item per gram tissue (1.0 g/tissue), the estimated mean human intake of microplastics for the general population consuming 50 g of the assessed species was 50 MP items/week or 2,600 MP items/year, corresponding to 1.0 MP item/g/week and 52 MP item/g/year. These figures are many folds higher than those previously estimated for humans consuming oysters from South Korea, namely 0.412 MP items/week and 21.4 MP items/year, corresponding to 0.071 MP items/g/week and 3.70 MP items/g/year (Cho et al., 2019) and *Crassostrea gigas* from France namely 21 MP items/week or 1,136 MP items/year, corresponding to 0.45 MP items/g/week and 28.5 MP items/g/year (Van Cauwenberghe & Janssen, 2014).

Our findings suggest that the annual dietary exposure for Ghanaian shellfish consumers might be one order of magnitude higher. Despite the high level of human exposure with reference to this study, uncertainties remain; it is unknown if the ingested dose equals the absorbed dose or whether cooking significantly affects plastic toxicity. Although the study provides baseline data on the occurrence and abundance of microplastics in shellfish from the coastal waters of Ghana, we acknowledge that the sample size is relatively small and so it is recommended that further studies are undertaken on a wider scope.

## 5 Conclusion

This study provides baseline data on microplastics in oysters from the coastal waters of Ghana. Oysters and other species of bivalves are classified globally as good indicators of pollutants and this study has confirmed that the oysters may be considered in future studies as a potential candidate for plastic polymer monitoring in West Africa where they predominate. Microplastic abundance was high in oysters from populated communities due to intensive anthropogenic activities. The high prevalence of microfibers in oyster samples suggests improper disposal or mismanagement of effluent into water bodies. Our findings further indicate that microplastic abundance in oysters does not depend on the size of the organism but rather on the time and frequency of exposure. The estimated microplastic intake per capita per year for Ghana revealed that exposure to microplastics from shellfish consumption may be significantly higher in countries with high oyster consumption rates. However, the toxic effect of microplastics on human health is not well understood. Therefore, further studies are needed to highlight the toxicity of plastic exposure to human health.

## Declarations

### Author contribution statement

Samuel Addo: Conceived and designed the experiments; Contributed reagents, materials, analysis tools or data; Wrote the paper.

Charles Mario Boateng; Harriet Danso - Abbeam: Analyzed and interpreted the data; Wrote the paper.

Rhode lims Diyie: Conceived and designed the experiments; Analyzed and interpreted the data.

Collins Prah Duodu: Performed the experiments; Analyzed and interpreted the data; Wrote the paper.

Kofi Ferni Anyan: Performed the experiments; Analyzed and interpreted the data.

Ernestina Abbew Williams; Akosua Ohemaa Amakye: Performed the experiments.

Obed Asamoah; Elvis Nyarko: Analyzed and interpreted the data.

### Funding statement

This research did not receive any specific grant from funding agencies in the public, commercial, or not-for-profit sectors.

### Data availability statement

Data will be made available on request.

### Declaration of interest’s statement

The authors declare no conflict of interest.

### Additional information

No additional information is available for this paper.
